# DCE-MRI of patient-derived xenograft models of uterine cervix carcinoma: associations with parameters of the tumor microenvironment

**DOI:** 10.1186/s12967-017-1331-4

**Published:** 2017-11-03

**Authors:** Anette Hauge, Catherine S. Wegner, Jon-Vidar Gaustad, Trude G. Simonsen, Lise Mari K. Andersen, Einar K. Rofstad

**Affiliations:** Group of Radiation Biology and Tumor Physiology, Department of Radiation Biology, Institute for Cancer Research, The Norwegian Radium Hospital, Oslo University Hospital, P. O. Box 4953 Nydalen, 0424 Oslo, Norway

**Keywords:** Cervical carcinoma, PDX models, DCE-MRI, Tumor microenvironment, Hypoxia

## Abstract

**Background:**

Abnormalities in the tumor microenvironment are associated with resistance to treatment, aggressive growth, and poor clinical outcome in patients with advanced cervical cancer. The potential of dynamic contrast-enhanced (DCE) MRI to assess the microvascular density (MVD), interstitial fluid pressure (IFP), and hypoxic fraction of patient-derived cervical cancer xenografts was investigated in the present study.

**Methods:**

Four patient-derived xenograft (PDX) models of squamous cell carcinoma of the uterine cervix (BK-12, ED-15, HL-16, and LA-19) were subjected to Gd-DOTA-based DCE-MRI using a 7.05 T preclinical scanner. Parametric images of the volume transfer constant (*K* ^trans^) and the fractional distribution volume (*v*_e_) of the contrast agent were produced by pharmacokinetic analyses utilizing the standard Tofts model. Whole tumor median values of the DCE-MRI parameters were compared with MVD and the fraction of hypoxic tumor tissue, as determined histologically, and IFP, as measured with a Millar catheter.

**Results:**

Both on the PDX model level and the single tumor level, a significant inverse correlation was found between *K* ^trans^ and hypoxic fraction. The extent of hypoxia was also associated with the fraction of voxels with unphysiological *v*_e_ values (*v*_e_ > 1.0). None of the DCE-MRI parameters were related to MVD or IFP.

**Conclusions:**

DCE-MRI may provide valuable information on the hypoxic fraction of squamous cell carcinoma of the uterine cervix, and thereby facilitate individualized patient management.

**Electronic supplementary material:**

The online version of this article (10.1186/s12967-017-1331-4) contains supplementary material, which is available to authorized users.

## Background

Despite recent advances in prevention and screening of human papilloma virus (HPV)-associated malignancies, carcinoma of the uterine cervix remains a life-threatening disease world-wide. The majority of cervical cancer patients is diagnosed with squamous cell carcinoma, for which concurrent chemoradiotherapy is the first-line treatment option in advanced cases [1]. Clinical outcome is, to a great extent, affected by the presence of severe abnormalities in the microenvironment of the primary tumor, such as elevated interstitial fluid pressure (IFP), high lactate concentration, low oxygen tension, and large hypoxic regions [2–5]. In order for improved and more personalized therapeutic strategies to be developed, biomarkers reflecting the biological characteristics of the tumor tissue are needed.

Hypoxia has been associated with invasive growth, treatment resistance, and poor survival rates in many cancers, including cervical cancer [4–6]. Furthermore, both microvascular density (MVD) and IFP are thought to influence the metastatic propensity of solid tumors [2, 7, 8]. Accurate assessment of these critical parameters would therefore be of great prognostic value and facilitate clinical decision-making on an individual patient basis. As a noninvasive and commonly used method in the management of cervical cancer, dynamic contrast-enhanced (DCE) MRI is an attractive technique for this purpose. Preclinical studies on cell line-derived tumor xenografts have suggested that DCE-MRI-derived parameters may have the potential to serve as biomarkers for microenvironmental features of cervical cancer [9–12]. Promising observations have also been made in clinical settings [13–15], but further investigations are required to explore the true benefits of DCE-MRI for other purposes than anatomical characterization of the tumor tissue.

In order to evaluate the hypothesis that DCE-MRI can provide clinically useful information about the tumor microenvironment, patient-derived xenograft (PDX) models were utilized in this study. Compared to the cell line-derived tumor models used in most preclinical in vivo studies of cervical cancer, PDX models are thought to mirror the biological characteristics of patient tumors to a larger extent [16, 17]. However, as the tumors of different patients within the same prognostic group tend to display substantial heterogeneity, a panel of PDX models is typically required for clinical issues to be addressed. Hence, four biologically dissimilar PDX models of squamous cell carcinoma of the uterine cervix were subjected to DCE-MRI in the present investigation. Imaging results were compared with findings from immunohistochemical examinations and IFP measurements, with the overall aim to reveal potential associations between DCE-MRI-derived quantities and parameters of the tumor microenvironment, both on the single tumor level and the PDX model level.

## Methods

### PDX models

Adult (8–12 weeks of age) female BALB/c *nu*/*nu* mice were used as host animals for xenografted tumors. Four PDX models (BK-12, ED-15, HL-16, and LA-19) of squamous cell carcinoma of the uterine cervix, obtained from patients with FIGO stage IIB disease prior to treatment, were included in the study [18, 19]. We have established two frozen cell stocks of these models, one from xenografted tumors in passage 2 (*early* generation) and the other from xenografted tumors transplanted serially in mice for approximately 2 years (*late* generation). Experiments were carried out with early generation as well as late generation xenografts. Tumors were initiated in the left *quadriceps femoris* of mice by inoculating aliquots of 5 × 10^5^ cells derived from intramuscular tumors initiated from the frozen stocks, and they were included in experiments when having grown to a volume of 100–1600 mm^3^.

### Magnetic resonance imaging

A Bruker Biospec 7.05 T bore magnet, equipped with a mouse quadrature volume coil (Bruker Biospin, Ettlingen, Germany), was used for MR imaging. Early and late generation tumors (9–16 in number) of each PDX model were imaged with axial slices covering their entire volume. Mice were anesthetized with 0.5 l/min O_2_ containing ∼ 4.0% Sevofluran (Baxter, Illinois, USA) during scanning. Respiration rate and body core temperature were monitored continuously with a pressure sensitive abdominal probe and a rectal temperature probe (Small Animal Instruments, New York, NY, USA). Automated hot air flow regulation kept the body core temperature at 37 °C, while a stable respiration rate was maintained by manual adjustment of the gas anesthesia.

Anatomical *T*_2_-weighted images were obtained with a fast spin echo pulse sequence (RARE) with a repetition time (TR) of 2500 ms, an echo time (TE) of 35 ms, an image matrix of 128 × 128, a field of view (FOV) of 3 × 3 cm^2^, a slice thickness of 0.7 mm, a slice gap of 0.3 mm, two averages, and fat suppression. DCE-MRI was performed as previously reported [20]. In short, precontrast *T*_1_ values (*T*_10_ values) were measured by applying a fast spin echo pulse sequence (RARE) with TRs of 200, 400, 800, 1500, 3000, and 5000 ms, a TE of 8.5 ms, an image matrix of 128 × 128, a FOV of 3 × 3 cm^2^, a slice thickness of 0.7 mm, and a slice gap of 0.3 mm. Gd-DOTA (Dotarem, Guerbet, Paris, France), diluted to a final concentration of 0.06 M, was used as contrast agent. With an automated infusion pump (Harvard Apparatus, Holliston, MA, USA), Gd-DOTA was injected into the tail vein of mice in a bolus dose of 5.0 ml/kg during a period of 5 s. Dynamic *T*_1_-weighted images were recorded at a temporal resolution of 14.8 s, using a three-dimensional SPGR pulse sequence (3D-FLASH) with a TR of 10 ms, a TE of 2.07 ms, a flip angle of 20°, an image matrix of 128 × 128 × 10, and a FOV of 3 × 3 × 1 cm^3^.

The DCE-MRI series were analyzed using regions of interest (ROIs) encompassing the tumor tissue. ROIs were delineated in the *T*_2_-weighted anatomical images acquired prior to DCE-MRI. For each voxel, numerical values of the volume transfer constant (*K* ^trans^) and the fractional distribution volume (*v*_e_) of Gd-DOTA were calculated by a best curve fit approach utilizing the Tofts pharmacokinetic model [21], with the arterial input function of Benjaminsen et al. [22]. Calculation of Gd-DOTA concentrations and pharmacokinetic modeling were performed with in-house-made software developed in Matlab (MathWorks, Natick, MA, USA). Voxels assigned unphysiological *v*_e_ values (*v*_e_ > 1.0) were excluded from further quantitative analysis, in which whole tumor median values of *K* ^trans^ and *v*_e_ were considered.

### Histological examination

Histological sections, prepared by standard procedures, were stained with hematoxylin and eosin (HE) or immunostained for blood vessels or hypoxia. CD31 was used as a marker of endothelial cells, and pimonidazole [1-[(2-hydroxy-3-piperidinyl)-propyl]-2-nitroimidazole] was used as a marker of tumor hypoxia [23]. An anti-mouse CD31 rabbit polyclonal antibody (Abcam, Cambridge, UK) or an anti-pimonidazole rabbit polyclonal antibody (Professor Raleigh, University of North Carolina, Chapel Hill, NC, USA) served as primary antibody. Diaminobenzidine was utilized as chromogen, and hematoxylin was used for counterstaining. For each immunostaining, three sections cut through central regions of every tumor were analyzed. Microvessels were identified and scored manually as number of vessels per mm^2^ of non-necrotic tissue [24]. The hypoxic fraction, defined as the area fraction of viable tissue staining positive for pimonidazole, was determined by image analysis [25]. For each of the four PDX models, MVD was assessed in 20 early generation tumors, whereas hypoxia was quantified in 12–28 early and late generation tumors.

### Interstitial fluid pressure

IFP was measured with a Millar SPC 320 catheter equipped with a 2F Mikro-Tip transducer (Millar Instruments, Houston, TX, USA) [26]. A Millar TC-510 control unit and a preamplifier connected the catheter to a computer, and the LabVIEW software (National Instruments, Austin, TX, USA) was used for data acquisition. Two IFP measurements were performed for each of 10–20 early generation tumors of each PDX model, with the probe inserted in central tumor regions. The mean of the two IFP values was included in the subsequent analyses.

### Statistical analysis

The SigmaStat statistical software package (Systat Software Inc., San Jose, CA, USA) was utilized for statistical analysis. Correlations between parameters were searched for using the Pearson product moment correlation test. Regression analysis was carried out to characterize parameter relationships. Probability values of *P* < 0.05 were considered statistically significant.

## Results

### Histological appearance

**Fig. 1:**
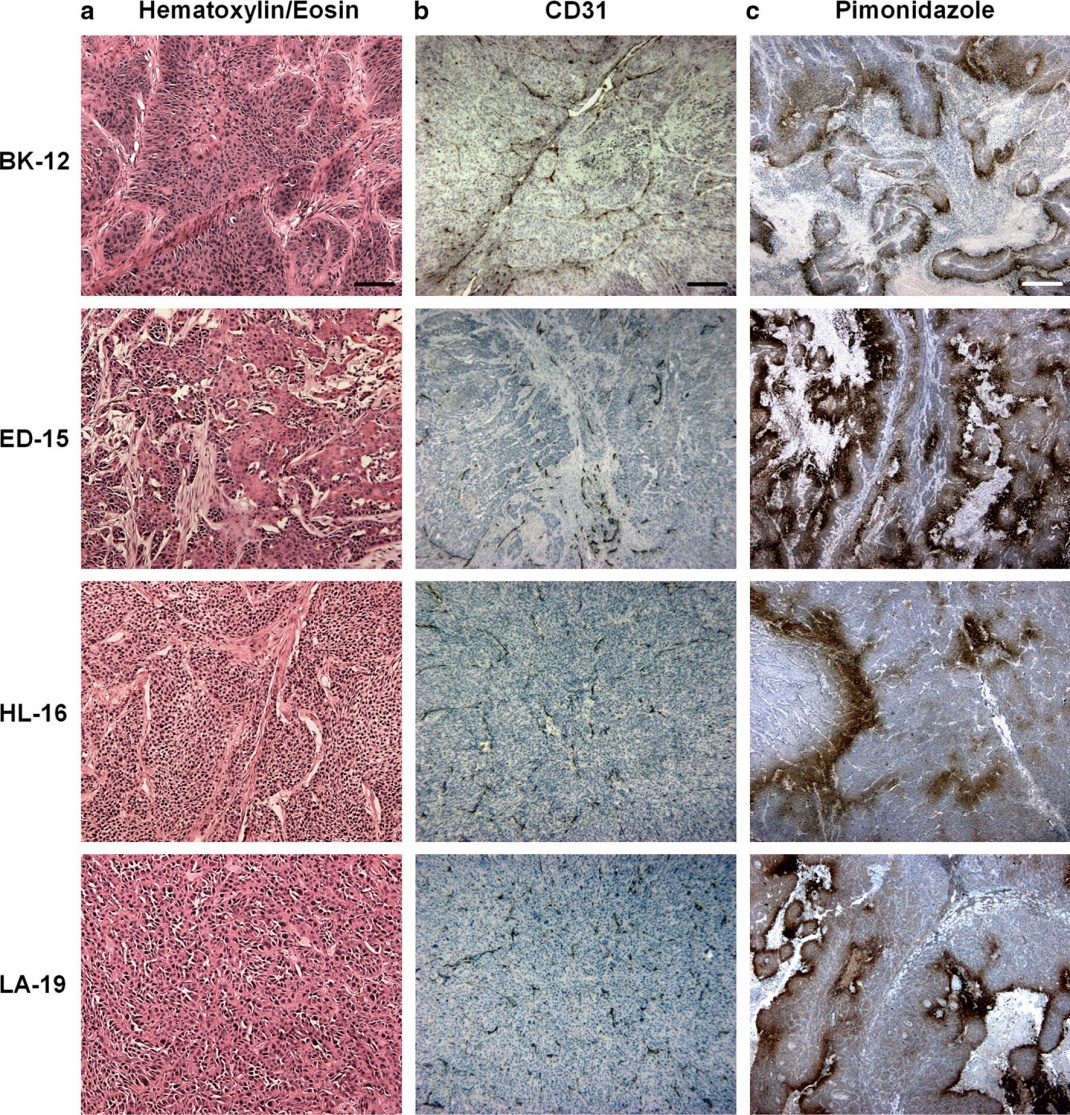
Histological preparations of a BK-12, ED-15, HL-16, and LA-19 tumor, stained with hematoxylin and eosin (**a**), immunostained for CD31 (**b**), and immunostained with an anti-pimonidazole antibody for hypoxia (**c**). Scale bars: 100 μm (**a**), 250 μm (**b**), and 500 μm (**c**)

The histological appearance of the four PDX models differed notably (Fig. 1). While BK-12, ED-15, and LA-19 tumors were moderately differentiated, tumors of the HL-16 model were poorly differentiated. CD31-positive vessels were located in central as well as peripheral tumor regions in all four models, and the vessel density increased from the tumor center towards the tumor periphery. Tissue staining positive for pimonidazole appeared as scattered foci of different size and shape in non-necrotic areas, or as a few cell layers thick rim encompassing necrotic regions. Both staining patterns were characteristic features of BK-12 and ED-15 tumors. In HL-16 tumors, little necrosis was detected, and focal staining constituted most of the regions defined as hypoxic. LA-19 tumors, on the other hand, showed large necrotic areas, and were dominated by a perinecrotic staining pattern. Early and late generation tumors of the same PDX model were qualitatively similar in terms of histological features and hypoxia staining.

### DCE-MRI parameters

**Fig. 2:**
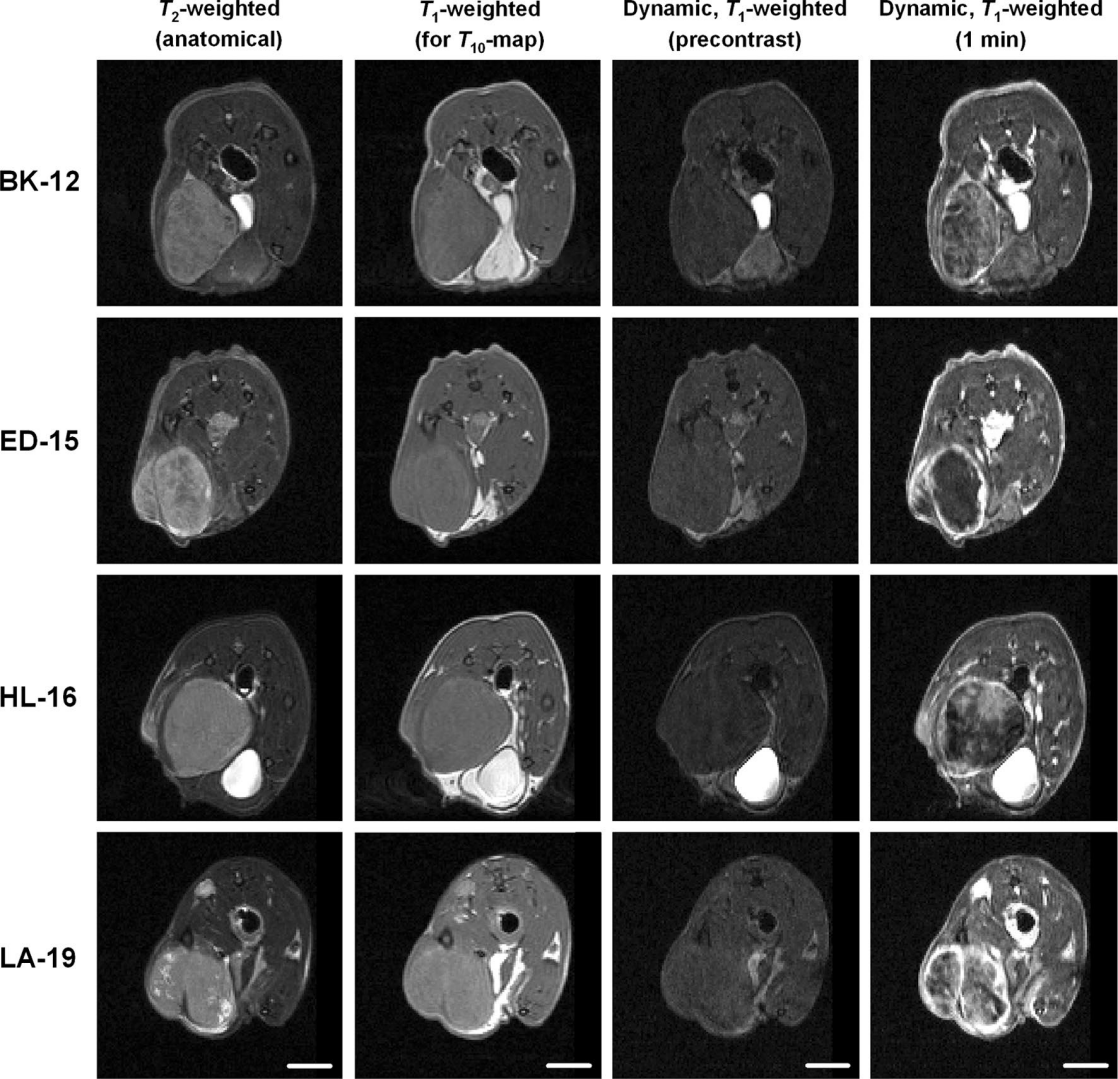
MR images of a representative BK-12, ED-15, HL-16, and LA-19 tumor. Displayed from left to right are anatomical *T*_2_-weighted images, *T*_1_-weighted images acquired for calculation of precontrast *T*_10_ values, and *T*_1_-weighted DCE-MR images recorded before and 1 min after the administration of Gd-DOTA. Scale bars: 5 mm

**Fig. 3:**
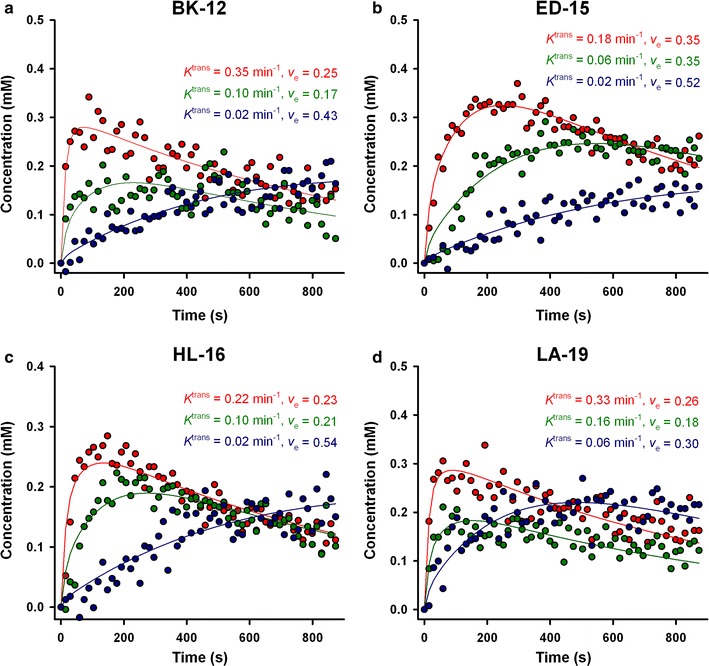
Concentration of Gd-DOTA versus time (dots) and the corresponding pharmacokinetic curve fit (solid lines) for individual voxels of a representative BK-12 (**a**), ED-15 (**b**), HL-16 (**c**), and LA-19 (**d**) tumor. The Tofts pharmacokinetic model was used to determine the value of *K* ^trans^ and *v*_e_ for each voxel

**Fig. 4:**
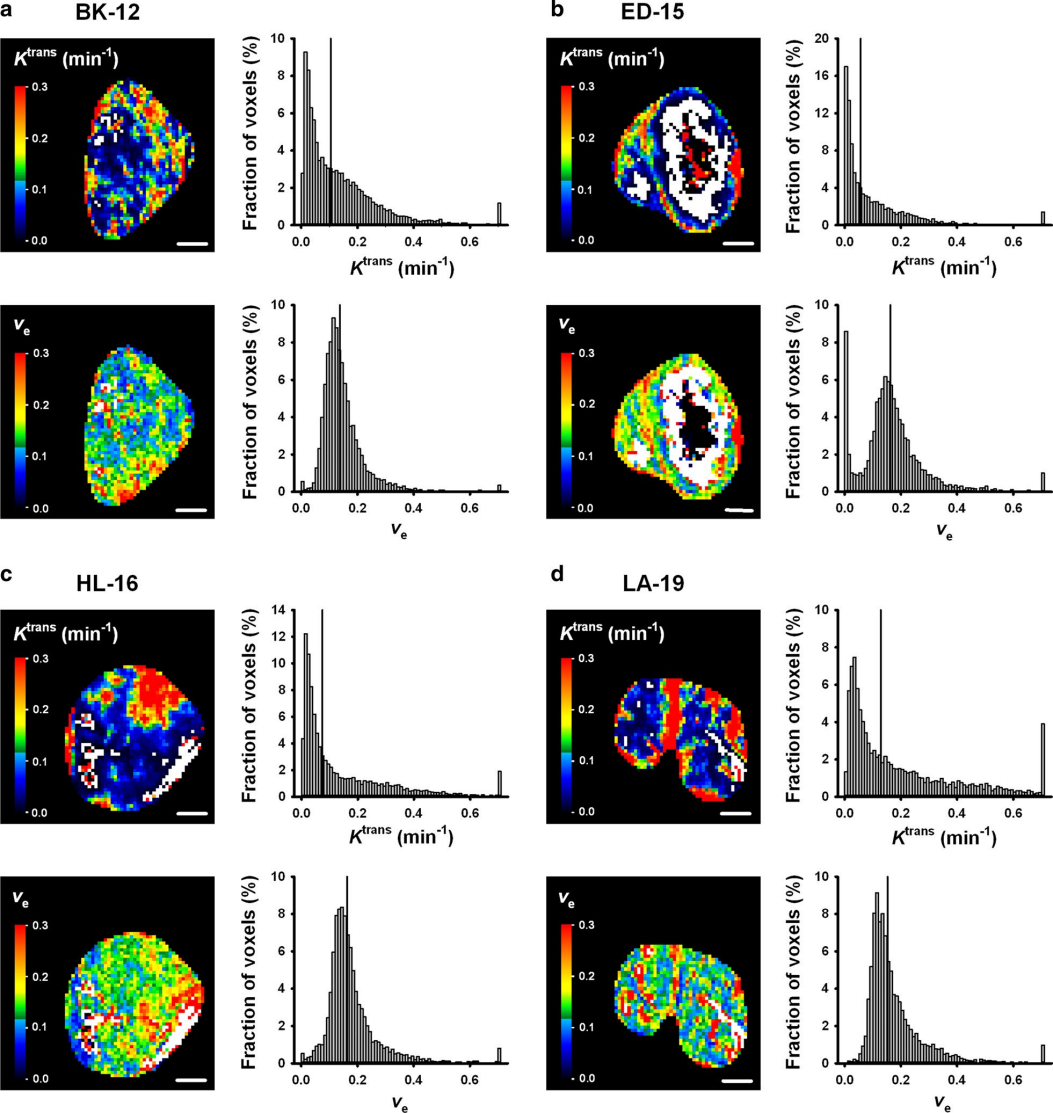
*K* ^trans^ and *v*_e_ images and frequency distributions of a representative BK-12 (**a**), ED-15 (**b**), HL-16 (**c**), and LA-19 (**d**) tumor. A central axial tumor section is shown in the parametric images, while individual voxel values from all tumor sections are included in the frequency distributions. Voxels with *v*_e_ > 1.0 (*white* in parametric images) have been excluded. Tumor median values are indicated by vertical lines. Color bars: *K* ^trans^ or *v*_e_ scales. Scale bars: 2 mm

MR images of a representative tumor of each xenograft model are presented in Fig. 2. Anatomical *T*_2_-weighted images, *T*_1_-weighted images needed for calculation of precontrast *T*_10_ values, and dynamic *T*_1_-weighted images recorded prior to and 1 min after contrast administration have been included. The standard Tofts model yielded good fits to experimental data for individual voxels with highly different uptake of the contrast agent, as illustrated by the representative plots of Gd-DOTA concentration versus time and the associated curve fits displayed in Fig. 3. *K* ^trans^ and *v*_e_ images and frequency distributions for the same tumors as shown in Fig. 2 are presented in Fig. 4. Voxels with unphysiological *v*_e_ values (*v*_e_ > 1.0) are *white* in parametric images and have been excluded from frequency distributions. Corresponding parametric images and frequency distributions prior to removal of unphysiological voxels are provided in Additional file 1: Figure S1. Tumor heterogeneity was considerable, both among tumors of the same PDX model and within individual tumors. On the single tumor level, *K* ^trans^ values had a tendency to be higher towards the periphery of the tumor tissue, whereas voxels with high *v*_e_ values were distributed more evenly throughout the tumor volume or clustered closer to central regions. Qualitative differences between early and late generation xenografts of the same tumor model were not detectable.

### Correlations between parameters

**Fig. 5:**
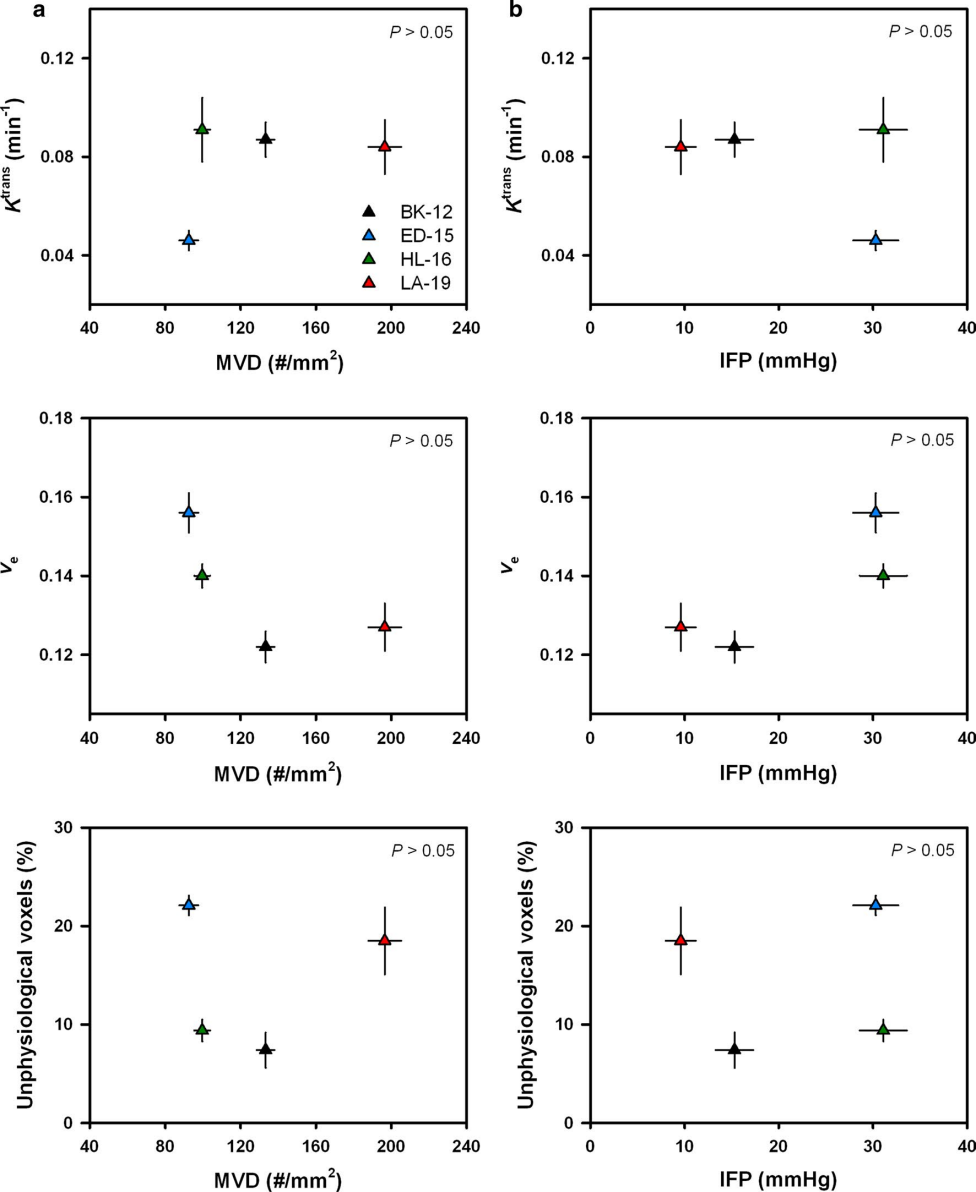
Median *K* ^trans^, median *v*_e_, and the fraction of unphysiological voxels versus microvascular density (MVD) (**a**) and interstitial fluid pressure (IFP) (**b**) for early generation tumors. Symbols and bars represent mean values ± SEM of 9–20 tumors

**Fig. 6:**
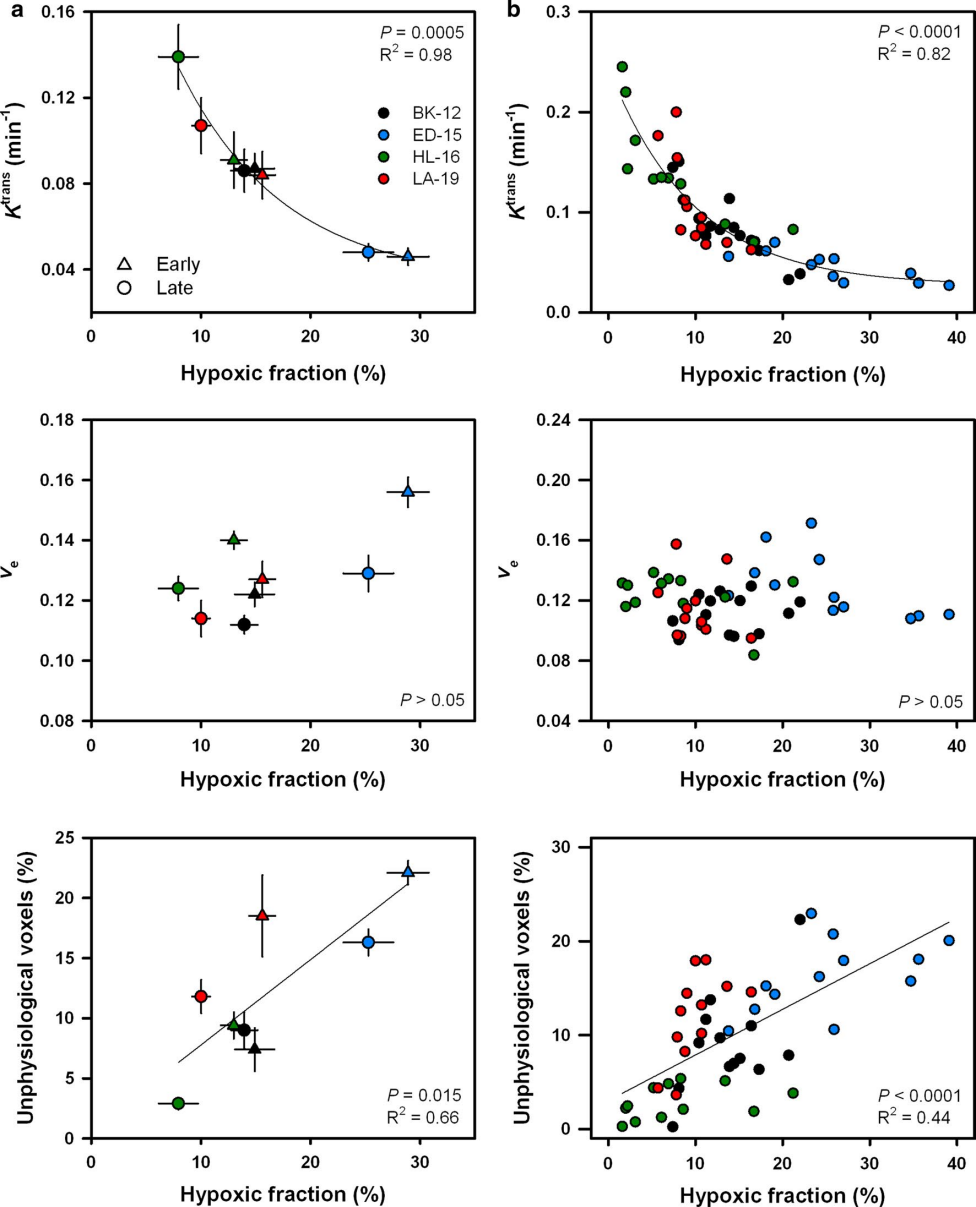
Median *K* ^trans^, median *v*_e_, and the fraction of unphysiological voxels versus hypoxic fraction for early generation (triangles) and late generation (dots) tumors. **a** Symbols and bars represent mean values ± SEM of 9–28 tumors. **b** Symbols represent individual tumors. Solid lines are regression curves

MRI and histology examinations were performed on different cohorts of mice for early generation xenografts, whereas the data for late generation xenografts were derived from the same tumors. Figure 5 presents quantitative relationships between DCE-MRI parameters and MVD and IFP for early generation tumors of each PDX model. As depicted, no statistically significant associations were found to be present. Figure 6 displays quantitative relationships between DCE-MRI parameters and the extent of hypoxia. In Fig. 6a, median *K* ^trans^, median *v*_e_, and the fraction of unphysiological voxels are plotted as a function of hypoxic fraction for early and late generation tumors. A significant correlation was found between *K* ^trans^ and hypoxic fraction (*P* = 0.0005), a correlation that was well described by an exponential decay curve (*y* = *y*_*0*_ + *ae* ^−*bx*^). No association was detected between *v*_e_ and the level of hypoxia, whereas there was a statistically significant linear correlation between the fraction of unphysiological voxels and hypoxic fraction (*P* = 0.015). Individual late generation tumor values are presented in Fig. 6b. On the single tumor level, as on the PDX model level, a significant relationship was found between *K* ^trans^ and hypoxic fraction (*P* < 0.0001), and between the fraction of unphysiological voxels and hypoxic fraction (*P* < 0.0001).

## Discussion

The potential of DCE-MRI to provide clinically valuable information on the tumor microenvironment has been investigated in numerous studies, both preclinical and clinical. On the clinical side, significant correlations between DCE-MRI-derived parameters and microvascular density, IFP, and oxygen tension have been reported [14, 15, 27], as well as promising findings on the ability of semi-quantitative and quantitative DCE-MRI measures to predict response to therapy [13, 28, 29]. Nevertheless, correlations have in general been relatively weak, and semi-quantitative DCE-MRI analyses that are hard to standardize remain to be preferred over more complex and labor intensive quantitative analyses in clinical practice.

A major advantage of preclinical studies compared to clinical studies in imaging-based research, is that the imaging conditions can be controlled more easily. High-quality preclinical investigations may therefore be a prerequisite for the true value of imaging-based approaches to be explored. Moreover, as tumors of the same genetic basis can be established in several animals, multiple copies of a single patient tumor can be examined in the same experiment. These tumor copies will inevitably differ somewhat in vascular density, interstitial hypertension, and the level of hypoxia, as a result of the stochastic nature of angiogenesis and differences in lesion size [30]. They may therefore, collectively, depict some of the intratumor heterogeneity characteristic of many human tumors.

Previous DCE-MRI studies of human cervical cancer xenografts in mice have revealed robust correlations between pharmacokinetic parameters and IFP or the extent of hypoxia, i.e. the fraction of radiobiologically hypoxic cells or the fraction of hypoxic tissue as determined histologically [9, 10, 12]. A possible drawback of these encouraging preclinical investigations is, nonetheless, the use of xenografted tumors initiated from previously established cell lines. Increasing evidence suggests that cell line-derived tumor xenografts may be suboptimal models of human cancer, primarily due to their limited capability of recapitulating the biological characteristics and diversity of patient tumors, as well as their lack of success in predicting clinical treatment outcome [16]. In this regard, PDX models, established by transplanting samples of patient tumors directly into host animals and maintaining the tumor tissue exclusively in vivo, have shown promise [16, 17, 31]. Our group has developed a panel of four PDX models of squamous cell carcinoma of the uterine cervix, differing in molecular and biological properties. Despite being intramuscular and thus ectopic tumor models, previous validation of the BK-12, ED-15, HL-16, and LA-19 tumors has confirmed that essential features of the donor patients’ tumors are retained after xenotransplantation [18, 19]. Furthermore, the highly different MVDs, IFPs, and fractions of hypoxic tissue found among these tumor xenografts make them suitable models for evaluating the hypothesis of investigation in the present study. It should be noted, however, that since the stroma and hematopoietic system in xenografted tumors is of murine origin, the vascular density, IFP, and supply of oxygen may deviate from that in human tumors [16].

The Tofts pharmacokinetic model was used to analyze the imaging data acquired in this study. Among other frequently used pharmacokinetic models, e.g. the Brix model and the shutter-speed model, the standard Tofts model has been reported to be preferable in analysis of clinical DCE-MRI data [32]. Previous investigations in our group have shown that good fits to experimental data are obtained using this model [20, 33], as well as highly reproducible parametric images [34]. Important prerequisites for the Tofts model, such as insignificant effects of water exchange, uniform concentration of the contrast agent within each voxel, and negligible contribution of intravascular contrast agent to the total tumor concentration, were assumed to be adequately fulfilled also in the present study. Although variation in arterial input function between animals was not considered, this pharmacokinetic model appeared to be well suited for analyzing the current DCE-MRI data. Nevertheless, in all four PDX models, a sizeable fraction of the voxels was assigned unphysiological *v*_e_ values, i.e. a fractional distribution volume of the contrast agent higher than unity. These voxels are likely to represent necrotic or fibrotic tissue, for which the Tofts pharmacokinetic model breaks down [35]. Consequently, voxels with *v*_e_ > 1.0 were excluded from the subsequent analysis. Exclusion of voxels with unphysiologically *low v*_e_ values was also considered, but since removal of these voxels only had a minor impact on the tumor median *K* ^trans^ and *v*_e_ values, simplicity of the analysis was chosen over the introduction of more exclusion criteria. In the present investigation, positive correlations were detected between the fraction of unphysiological voxels and the extent of hypoxia, in accordance with the well-established fact that severe and prolonged hypoxia may lead to necrosis [36].

When the basic assumptions of the Tofts pharmacokinetic model are valid, the volume transfer constant *K* ^trans^ is determined by the blood perfusion and the permeability surface area product of the vessel wall in varying proportions [37]. Considering the relatively high microvascular permeability in many cancerous tissues, as well as the low molecular weight of Gd-DOTA, *K* ^trans^ is thought to be largely dependent on blood perfusion in our DCE-MRI examinations of cervical cancer. Both on the PDX model level and the single tumor level, we found significant correlations between *K* ^trans^ and hypoxic fraction. Furthermore, the data were well described by common exponential decay curves, and comprised both early and late generation tumor xenografts. The indication that DCE-MRI can provide reliable information on the extent of hypoxia therefore holds true across varying transplantation conditions and several patient-derived xenograft models differing in biological features.

Hypoxia is caused by an imbalance between oxygen supply and oxygen consumption [38]. The oxygen supply is governed by the blood perfusion, whereas the oxygen consumption depends on the respiratory activity of the tissue and, consequently, the density of cells. Hypoxic regions are therefore expected to coincide with areas characterized by poor blood perfusion and/or low extracellular volume fraction. No association was found between *v*_e_ and the hypoxic fraction in this study, suggesting that the level of hypoxia is determined primarily by the blood perfusion, i.e. the extent to which a functional vascular network is present, in our cervical cancer models. Also, an abnormal microvasculature, with a varying fraction of non-perfused tumor vessels, can help explain why no correlation was seen between *K* ^trans^ and MVD in these experiments. *K* ^trans^ quantifies the functioning of perfused vessels, while the CD31 analysis incorporates all microvessels, both perfused and non-perfused.

As mentioned introductorily, elevated IFP is a common feature of malignant lesions [39]. Fluid is forced out of tortuous and leaky tumor vessels by the hydrostatic microvascular pressure, and accumulates in the tumor interstitium due to impaired lymphatic drainage [40]. In the present investigation, none of the DCE-MRI parameters were related to IFP, implying that neither the blood supply nor the extravascular extracellular volume fraction is determinative for the interstitial hypertension in these tumors.

Former characterization of BK-12, ED-15, HL-16, and LA-19 tumors has revealed fairly large fractions of stroma (∼ 20–35%) in these PDX models [18]. A densely structured extracellular matrix could possibly represent a barrier to the transvascular and interstitial transport of molecules [41], and one could therefore speculate whether the underlying assumptions of the Tofts pharmacokinetic model are violated when subjecting our cervical cancer models to DCE-MRI. However, the results reported here on the inverse association between *K* ^trans^ and hypoxic fraction are similar to previous findings on melanoma xenografts in our group [42, 43]. The PDX models of current interest differ significantly in histological appearance and stromal content from these melanoma xenografts, and it is thus possible that *K* ^trans^ may relate to tumor hypoxia in a qualitatively similar way for tumors showing substantial variation in extracellular composition.

## Conclusions

For clinicians to provide individual cancer patients with the optimal treatment and a precise prognosis, true knowledge on the tumor microenvironment would be of great value. In the present study, strong correlations were detected between *K* ^trans^ and the amount of hypoxic tumor tissue in PDX cervix carcinoma models, supporting the development and integration of this imaging method in clinical management of cervical cancer. Moreover, as hypoxia represents a major obstacle for the cure of a wide range of cancers, these results may prove to be valuable also for patients with other diagnoses than squamous cell carcinoma of the uterine cervix.

## Additional file

**Additional file 1: Figure S1.** DCE-MRI parameter maps and frequency distributions prior to exclusion of unphysiological voxels.

## Abbreviations

DCE-MRI: dynamic contrast-enhanced magnetic resonance imaging
FIGO: International Federation of Gynecology and Obstetrics
FLASH: fast low angle shot
FOV: field of view
Gd-DOTA: gadolinium-tetraazacyclododecanetetraacetic acid (gadoteric acid)
HE: hematoxylin and eosin
HPV: human papilloma virus
IFP: interstitial fluid pressure
MVD: microvascular density
PDX: patient-derived xenograft
RARE: rapid acquisition with relaxation enhancement
ROI: region of interest
SPGR: spoiled gradient recalled
TE: echo time
TR: repetition time
