# Supplementary material for: DCE-MRI of patient-derived xenograft models of uterine cervix carcinoma: associations with parameters of the tumor microenvironment
Source: J Transl Med. 2017 Nov 3;15:225. doi: 10.1186/s12967-017-1331-4 (PMC5670634; doi:10.1186/s12967-017-1331-4)
Supplement: Supplementary file 1 — Additional file 1: Figure S1. DCE-MRI parameter maps and frequency distributions prior to exclusion of unphysiological voxels. [file 12967_2017_1331_MOESM1_ESM.pdf]

Additional file 1 to:

DCE-MRI of patient-derived xenograft models of uterine cervix carcinoma: associations with parameters of the tumor microenvironment

Anette Hauge[1] – anette.hauge@rr-research.no
Catherine S. Wegner[1] – catherine.sem.wegner@rr-research.no
Jon-Vidar Gaustad[1] – jon.vidar.gaustad@rr-research.no
Trude G. Simonsen[1] – trude.golimo.simonsen@rr-research.no
Lise Mari K. Andersen[1] – lise.mari.klepp.andersen@rr-research.no
Einar K. Rofstad[1]* – einar.k.rofstad@rr-research.no

[1]Group of Radiation Biology and Tumor Physiology, Department of Radiation Biology, Institute for Cancer Research, The Norwegian Radium Hospital, Oslo University Hospital, P. O. Box 4953 Nydalen, N-0424 Oslo, Norway

*Corresponding author

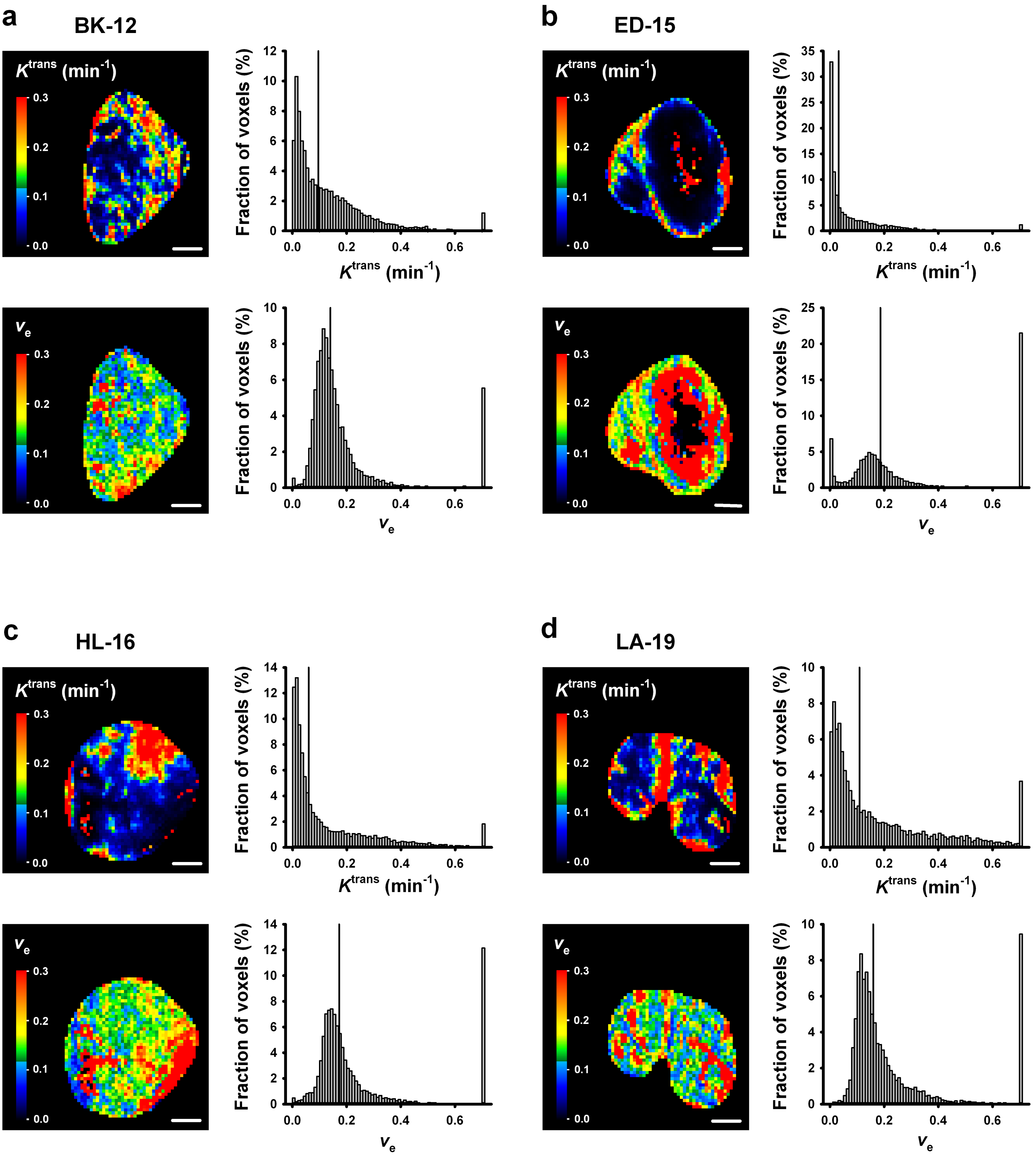

Figure S1. DCE-MRI parameter maps and frequency distributions prior to exclusion of unphysiological voxels.
K^{trans} and v_e images and frequency distributions of a representative BK-12 (**a**), ED-15 (**b**), HL-16 (**c**), and LA-19 (**d**) tumor. A central axial tumor section is shown in the parametric images, while individual voxel values from all tumor sections are included in the frequency distributions. Tumor median values are indicated by vertical lines.
Color bars: K^{trans} or v_e scales. *Scale bars:* 2 mm.
